# Marek’s disease virus replication in chicken skin reconstructed in vitro: evidence for viral particles in corneocytes

**DOI:** 10.1099/jgv.0.002123

**Published:** 2025-07-22

**Authors:** Laurent Souci, Mélanie Chollot, Katia Courvoisier-Guyader, Julia Lachner, Thibault Kervarrec, Julien Pichon, Julien Burlaud-Gaillard, Thibaut Larcher, David Pasdeloup, Leopold Eckhart, Caroline Denesvre

**Affiliations:** 1 INRAE-Université de Tours, UMR1282 ISP, Equipe Biologie des Virus Aviaires, Centre INRAE Val de Loire, Nouzilly, France; 2 Department of Dermatology, Medical University of Vienna, Vienna, Austria; 3 INRAE-Université de Tours, UMR1282 ISP, Equipe Biologie des Infections à Polyomavirus, Université de Tours, Tours, France; 4 INRAE-Université de Tours, UMR1282 ISP, Equipe Imagerie et Infectiologie, Centre INRAE, Nouzilly, Val de Loire, France; 5 EM Facility, Faculté de Médecine, Université de Tours, 10, Boulevard Tonnellé, Tours, France; 6 INRAE, Oniris, PAnTher, APEX, Nantes, France

**Keywords:** 3D *in vitro* models, chicken, herpesvirus, keratinocyte, Marek’s disease virus, viral particles

## Abstract

Marek’s disease (MD) is a lethal lymphoma of chickens, which is caused by MD virus (MDV), an alphaherpesvirus. MDV infects epithelial cells of the skin appendages, notably feather follicles, replicates in these cells and is shed into the environment exclusively from these tissues. Here, we tested whether chicken skin equivalents (SEs) can be used to model MDV infection. Primary chicken keratinocytes were seeded on a suspension of fibroblasts in collagen and induced to terminally differentiate at the air-liquid interface. A recombinant MDV expressing the Katushka fluorescent protein (MDV-KAT) was introduced into SEs by seeding primary keratinocytes together with MDV-KAT-infected keratinocytes of the K8 cell line. KAT-mediated fluorescence increased during the culture of infected SEs, indicating virus infection and replication, while the expression of keratinocyte differentiation markers was not significantly altered by MDV infection. MDV did not spread to the dermal compartment of SEs but localized to the upper layers of the epidermis. Viral particles were readily observed by electron microscopy in living keratinocytes and for the first time in cornified keratinocytes of the outermost layer of infected SEs, suggesting that viral elements can be released into the environment. Finally, we demonstrated that two fluorescent vaccine strains of MDV, Rispens and herpesvirus of turkey, can infect and replicate in SEs. Taken together, this study establishes chicken SEs as an *in vitro* model for essential steps of MDV infection.

## Introduction

Many avian viruses show a tropism for the skin or its appendages (reviewed in [1]). In contrast to mammal viruses, studying the interplay between avian viruses and the skin *in vitro* was limited to a single skin model: the skin explants (reviewed in [2]). This model is usable mostly to study pathogen entry after a wound. Recently, we published a protocol to reconstruct chicken skin *in vitro*, termed skin equivalent (SE), by a method similar to the ones used for humans or some other animals (reviewed in [2,4]). This method consists of cultivating primary keratinocytes on a dermis equivalent, made of fibroblasts in collagen, at an air-liquid interface (ALI) [5]. This *in vitro* model results in a multilayered stratified epithelium, with the basal layer, the suprabasal layers and the cornified layer, the outermost one [5]. This system recapitulates all steps of keratinocyte differentiation, from basal keratinocytes to corneocytes, the end product of terminal differentiation. Corneocytes are dead cells obtained through a process called cornification, in which organelles and nucleus are lost [6,7]. In mammals, such a model has proved to be of great interest to study virus interactions with the skin [8,11] as well as testing anti-viral drugs [8].

Marek’s disease (MD) is a lethal lymphoma of chickens caused by the highly transmissible Gallid alphaherpesvirus 2 (GaAHV-2), commonly known as MD virus (MDV) [12]. In addition to its tropism for lymphoid cells, this virus exhibits a strong tropism for skin epithelial cells [13]. Seven days post-infection, the virus starts to replicate in keratinocytes of feather follicles and of other hard skin appendages (i.e. scales that cover the legs) [14,15]. This results in virus shedding associated with skin dander and dust [16,20] and to durable contamination of the environment [18,21]. This material is the only known source of horizontal transmission between chickens in the field [17]. Like pathogenic MDV, MD vaccines such as Rispens strain (CVI988) and herpesvirus of turkey (HVT; meleagrid alphaherpesvirus 1) also infect and replicate in feather follicle epithelium (FFE) [22,26]. *In vivo*, the FFE is the only known site where fully infectious particles can be efficiently produced as cell-free virus and shed [14,17, 27, 28]. In this tissue, mature virions are visible by electron microscopy and exclusively in the intermediate layers of the epithelium [27,29].

Typically, both MDV and MD vaccines are produced *in vitro*, in primary chicken or duck embryonic fibroblasts cultured in monolayer [12], in which viruses remain strictly cell associated [30,32]. In these cells, very few mature MDV particles can be detected through electron microscopy transmission [28,33] and infectious virions cannot be purified from cell lysates. Consequently, viral infection is achieved through the cocultivation of infected cells with uninfected cells. Several years ago, we developed three chicken keratinocyte cell lines (K8, K1 and KP2) from chicken embryonic stem cells to refine MDV cell models [34]. These chicken keratinocytes are highly proliferative basal keratinocytes. While these keratinocytes, cultured as a monolayer, were permissive to MDV infection through cocultivation, the production of mature virions remained very low [35]. Because *in vivo*, mature virions have been seen only in suprabasal layers of FFE, we hypothesize that keratinocyte differentiation might be essential for the production of fully mature infectious MDV particles *in vitro* and/or shedding.

This study aims at evaluating the capacity of chicken three dimensional (3D) SE to support MDV infection and replication. We demonstrate that MDV efficiently infects and spreads in SE, as well as the vaccine viruses. MDV localizes in the upper layers of the reconstructed epidermis, notably in corneocytes. Interestingly, we also demonstrate that infection by MDV does not compromise SE reconstruction and keratinocyte differentiation.

## Methods

### Cell line and viruses

Primary chicken embryonic skin cells (CESCs) were prepared from 12-day-old embryonated eggs of specific-pathogen-free White Leghorn embryos, as described previously [36]. These cells were cultured in William’s modified E medium (#3772651, Fisher Scientific) supplemented with 2% chicken serum and 3% FCS, resulting in a population primarily composed of dermal fibroblasts, along with a few myoblasts and rare keratinocytes. The K8 cell line, a chicken keratinocyte line established from chicken embryonic stem cells, was cultivated and maintained according to earlier methods [34,35].

Three recombinant red fluorescent reporter viruses were used: the very virulent RB-1B TK Katushka MDV strain (MDV-KAT), the vaccinal CVI 988/Rispens mCherry (kind gift from Professor Venugopal Nair, Pirbright Institute, UK) and the HVT FarRed strain (kind gift from Dr Motoyuki Esaki, CEVA, Japan). Note that the HVT FarRed was not obtained from a bacterial artificial chromosome (BAC), unlike the two others, but by recombination in primary chicken fibroblasts. The MDV-KAT virus was generated using a BAC of the very virulent RB-1B strain [37] using the two-step Red-mediated recombination system as described previously [38] on the backbone of the rTK-GFP (kind gift of Dr V. Nair), with the Katushka fluorescent gene replacing the GFP gene. For that, the plasmid pKanInKatushka was designed by our laboratory and synthetized by GeneArt (Invitrogen) (sequence on demand). Sequences flanking the TK locus in the BACmid were directly incorporated within pKanInKatushka. Katusha primers were used to amplify the cassette and to monitor the insertion and subsequent removal of the cassette (Table 1). The recombinant virus was obtained after transfection of 5×10^6^ CESCs with 4 µg of the mutant BAC by electroporation using the Amaxa Nucleofector apparatus (programme 024) with the basic Nucleofector kit for primary mammalian fibroblasts (Lonza). After 4 days of incubation, viral plaques were obtained. The MDV-KAT was next passaged on CESCs no more than three times and titrated. All fluorescent viruses were produced in CESCs cultivated in William’s modified E medium supplemented with 1% chicken serum and 1.5% FCS.

**Table 1.** Primers and probes

**Name**	**Sense**	**Sequence (5′→3′)**
***Construction of MDV-KAT (Katushka primers)***	For	CACTTCGCATATTAAGGTGACACG
	Rev	ACAAGTTAACGTCGACCCGGGTAC
**TaqMan quantitative PCR**		
SORF1 HVT	For	GGCAGACACCGCGTTGTAT
	Rev	TGTCCACGCTCGAGACTATCC
	Probe	FAM-AACCCGGGCTTGTGGACGTCTTC-TAMRA
ICP4 MDV	For	TCTTGCACCGAGATGATCGAT
	Rev	AAAATACCATAGATTCGAGAGGTTCAG
	Probe	FAM-AAATCCACCCGTCGAGTCGCCC-TAMRA
iNOS	For	GAGTGGTTTAAGGAGTTGGATCTGA
	Rev	TTCCAGACCTCCCACCTCAA
	Probe	FAM-CTCTGCCTGCTGTTGCCAACATGC-TAMRA
***Reverse Transcriptase-quantitative PCR***		
RPS17	For	ACACCC GTC TGG GCAACGAC
	Rev	CCCGCTGGATGCGCTTCATC
KRT14	For	GCGAGGACGCCCACATCTCTTC
	Rev	TGAGCGCCATCTGCTCACGG
LOR1	For	CCACGAGTCATCTTCCCAGT
	Rev	AGACGAACCTCCTCCTCCTC
KRT9-L3	For	GGAGGAAGCTGCATCATTGG
	Rev	GGCTGATTTCCCTTTGGCAG
KRT78-L3	For	GTGTATGTGGCTCAGGAGGA
	Rev	TGTTTCTTTCTCAGTCGGCC

### Plaque size assays

The size of the plaques was determined as described previously [39]. CESCs were seeded in six-well plates and infected with 100 p.f.u. of indicated viruses. Four days later, cells were fixed with 4% paraformaldehyde, permeabilized, blocked and stained with a cocktail of monoclonal antibodies (anti-gB, ICP4 and VP22) and revealed with a goat-anti-mouse-labelled with AlexaFluor488 (Invitrogen). At least 50 randomly selected plaques were imaged on the green channel on an Axiovert 200 M inverted microscope (Zeiss) with a 5× fluar long-distance objective, using an AxioCam MRm charge-coupled device camera (Zeiss) and Axiovision LE64 software (release 4.9.1, Zeiss). The area of imaged plaques was determined manually using Axiovision software (Zeiss).

### Viral infection of K8 cells

Four hundred fifty thousand chicken K8 keratinocytes were infected by co-seeding with MDV-KAT-infected CESCs (5,000 p.f.u./well of a six-well culture plate). Infection was performed during 5 h at 37 °C in 1.5% chicken serum and 1% FCS Dulbecco's Modified Eagle Medium (DMEM)-F12 medium (#21331020, Gibco). Medium was next replaced with William’s E medium supplemented with 5% chicken serum and 5% FCS for an overnight incubation time and then maintained for 4 additional days in William’s E medium supplemented with 1.5% chicken serum and 1% FCS. A similar infection method was applied for HVT FarRed and Rispens mCherry viruses.

### Isolation of primary fibroblasts and keratinocytes from chicken skin

Chicken primary fibroblasts and keratinocytes were isolated from the skin as previously described [5], with few adaptations described below. Herein, the skin was dissected from the legs of specific-pathogen-free White Leghorn embryos of 19 days [provided by the infectiology platform (PFIE) of INRAe (Tours-France) (https://www6.val-de-loire.inrae.fr/pfie_eng/)] and not 1-day-old chicks. Skin fragments were disinfected with iodine, excised and then incubated for 3 h at 4 °C with 2 mg ml^−1^ thermolysin (#T7902-100MG, Sigma). The epidermis was carefully separated from the dermis. The epidermis was enzymatically digested for 7 min at 37 °C in a 0.05% trypsin-EDTA solution (#11580626, Gibco). The dermis was digested for 30 min at 37 °C in a collagenase mix (5 mM MgSO_4_ (#5886 7H20, Merck) with 1 mg ml^−1^ collagenase I (#C2674, Sigma), 0.5 mg ml^−1^ hyaluronidase (#H3506, Sigma) and 10 µg ml^−1^ Dnase I (#11284932001, Roche)). The epidermis provided embryonic chicken primary keratinocytes (CPKe), mostly basal, and the dermis provided primary dermal fibroblasts. Isolated cells were filtered successively over 100 and 40 µm cell strainers (Falcon). Cells were centrifuged for 7 min at 1,400 r.p.m., and the pellets were resuspended in their respective culture medium as follows. Viable CPKe were counted using a multimode multiplate reader (Tecan Spark®). CPKe were directly used for SE production.

Primary dermal fibroblasts were cultured in gelatin-coated flasks in a William’s E medium (#32551-020, Gibco) supplemented with 3% chicken serum, 2% FCS, 1% l-glutamine and 1% penicillin-streptomycin (#DE17-602E, Lonza). These cells were used after one or several passages for dermis equivalent production.

### Production of 3D SEs

**Fig. 1.**
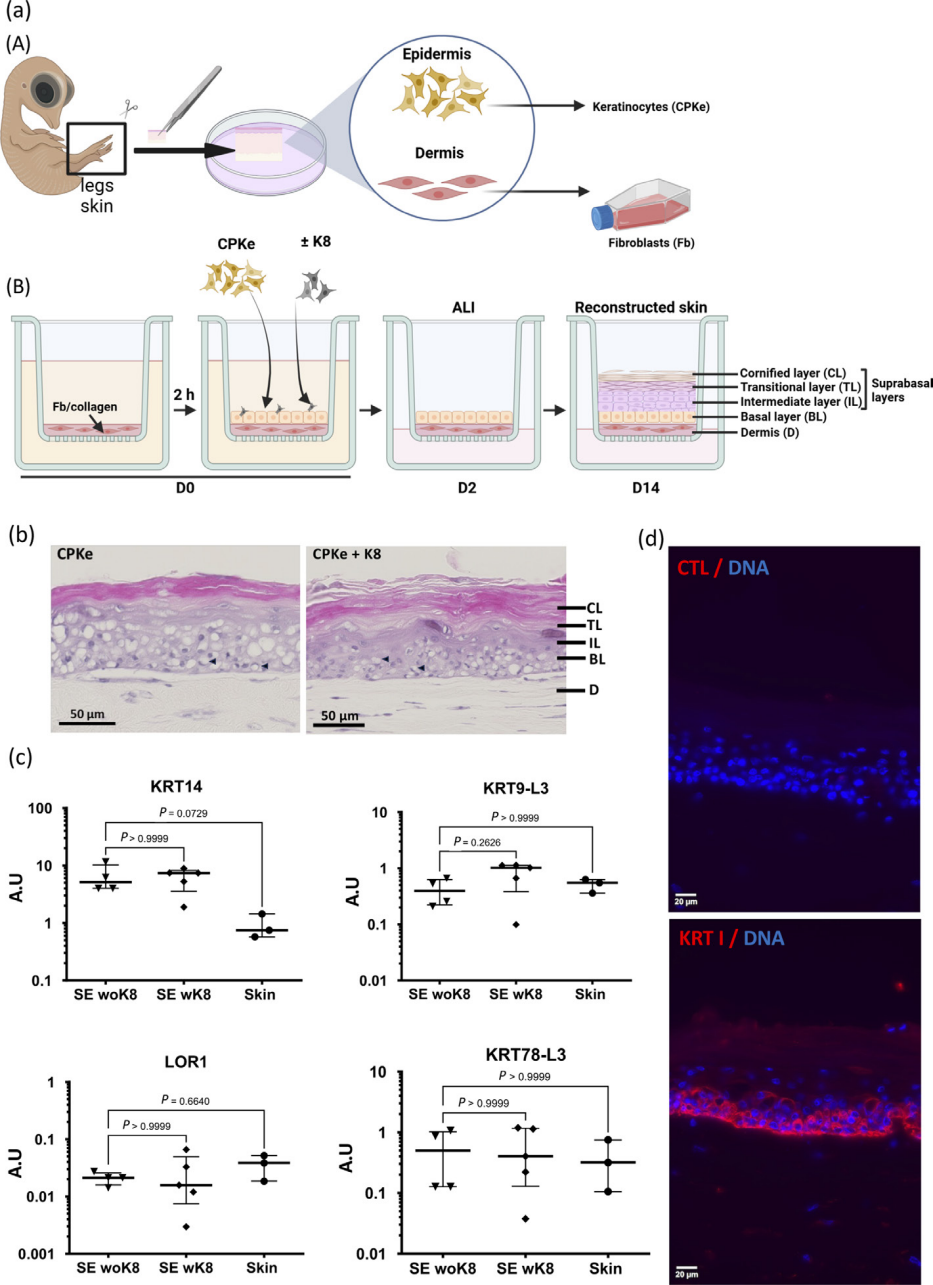
Validation of reconstructed chicken skin, named SEs, using chicken embryonic cells. (**a**) Schematic overview of the SE reconstruction. (A) CPKe and dermal fibroblasts were isolated, respectively, from the epidermis and the dermis of leg skin harvested from 19-day-old WL embryos. (B) SEs were produced in an insert, by using a fibroblast (Fb)-populated collagen matrix (salmon colour) on the top of which CPKe were seeded. Cells were cultured for 2 days immerged before being lifted at ALI. ALI facilitates keratinocyte differentiation and stratification into multiple layers, ultimately forming a cornified SE. The SE reconstruction was ended at 14 days post-seeding (D14). SE reconstruction was also performed by mixing K8 cells (a chicken keratinocyte cell line derived from chicken embryonic stem cells) with CPKe at the seeding step (D0). (**b**) Histological analysis. Images of haematoxylin-phloxin-safranin-stained sections of SE, performed with CPKe alone (left panel) or CPE supplemented with K8 (right panel). Small black arrowheads indicate vacuoles. (**c**) The transcript expression of skin differentiation markers in the SE was analysed by quantitative PCR, with chicken leg skin (noted skin on graphs), serving as control. In the graphs, each symbol represents independent samples (*n*=3 to 5). mRNA levels for each gene were normalized to the housekeeping gene RPS17, were expressed in arbitrary unit (A.U.) and were presented in a dot plot showing median values±interquartile range. The Kruskal–Wallis test with a Dunn correction was performed, using the chicken skin as a reference. Exact *P*-values are indicated on graphs. (**d**) Cryosections of SE with K8 were stained with an anti-KRT type I antibody (11E10) plus a secondary antibody labelled with Alexa Fluor 555. Nuclei (DNA) were stained with Hoechst 33342. Samples were observed by fluorescence microscopy. The red fluorescent signal indicated that KRT14 was localized in the lower layers of the epidermis (mostly basal layer). The nuclei are visible in the basal and suprabasal layers and very rarely not in the cornified layer.

Chicken SEs were reconstructed as previously described [5] with few modifications described below. Briefly, a collagen-fibroblast dermal equivalent was prepared using 8:10 (vol:vol) of 3.2 mg ml^−1^ bovine type I collagen, 1:10 (vol:vol) of Hanks Balanced Salt Solution (HBSS) 10X (#14060-040, Gibco), 1M NaOH (#58045-500 G, Sigma-Aldrich) to neutralize pH of collagen-HBSS mix and 1:10 (vol:vol) of dermal fibroblasts (250,000 cells) resuspended in chelated chicken serum. The solution was poured into cell culture inserts (#353092, Falcon), placed into six deep-well plates (#355467, Corning), let solidified for 2 h (37 °C without CO_2_) and finally incubated for 2 additional hours, submerged, in Keratinocyte Growth Medium 2 (KGM-2, #C20211, Promocell). Next, 2×10^6^ CPKe, freshly isolated, were seeded onto the dermal equivalent and cultured in 10% chicken keratinocytes medium instead of the KGM-2 medium used earlier [5]. The chicken keratinocyte medium consists of a 1:3 (vol:vol) Ham-s F12 (#21765-029, Gibco)/DMEM (#31053-028, Gibco) mixture supplemented with 10% calcium-chelated chicken serum (#16110-082, Gibco), 5 µg ml^−1^ insulin-transferrin-selenium (#51300044, Gibco), 10^–4^ M adenine (#A9795, Sigma), 10^–10^ M cholera toxin (#C8052, Sigma), 0.5 µg ml^−1^ hydrocortisone (#H0396, Sigma), 1.37 ng ml^−1^ triiodo-l-thyronine (#T6397, Sigma), 10 ng ml^−1^ recombinant hEGF (#236-EG, R and D System), 1.15 g l^−1^ glucose (#G8644, Sigma), 1% l-glutamine (#25030-024, Gibco) and 1% penicillin-streptomycin (#DE17-602E, Lonza). The inserts were subsequently incubated at 37 °C in CO_2_ incubator. The day of seeding is named here day 0 (‘D0’). Two days later (D2), the 3D skin cultures were lifted to ALI. At that time, skin keratinocyte differentiation medium supplemented with 0.1% BSA, 50 µg ml^−1^ ascorbic acid and 1.3 mM CaCl2 was used in the bottom compartment of the insert like in [5]. From there, SEs were cultured at ALI for 12 days (D14), with medium renewal in the bottom compartment every 2 days. Note that at the time of CPKe seeding, 0.5×10^6^ K8 cells infected or not with the virus were or were not added (see below). The complete process is schematically depicted in Fig. 1a. Thirty-three SEs from ten different batches were used in this study.

### Infection of SEs with virulent MDV and vaccine viruses

The SEs were infected during the 3D skin fabrication process, at D0. To proceed, K8 cells infected with one of the fluorescent viruses (MDV-KAT, Rispens mCherry or HVT FarRed) were trypsinized and resuspended in chicken keratinocyte medium (described above). For each insert, 0.5×10^6^ infected K8 cells were combined with 2×10^6^ CPKe and seeded onto the dermis equivalent. The SEs were then produced as previously described above. The analyses of the infected SEs were conducted at D14 post-infection, marking the endpoint of SE reconstruction, as well as at earlier time points when kinetics were performed (D2, D6, D9 and/or D12).

### SE imaging using a stereomicroscope

Freshly collected SEs were imaged using a Leica fluorescent stereomicroscope MZ10F. Images were captured with the DFC3000 monochrome camera (Leica, Nussloch, Germany) by using the LAS X software (Leica). Red fluorescence and/or white light exposure was set to a fixed exposure time with a fixed gain, in each independent experiment, in order to monitor the evolution of fluorescence intensity over time (e.g. 250 ms with a gain of 1.5).

Fluorescence intensity analysis was conducted using the FIJI software (v1.54f, available at https://imagej.net/software/fiji/downloads) on one to three MDV-KAT-infected SEs. Briefly, a region of interest (ROI) matching the dimensions of the sample was manually defined on the images, and the average fluorescence intensity within this ROI was subsequently measured.

### Histology

SEs were fixed with 4% formalin, embedded in paraffin wax, from which 4 µm sections were cut and stained using haematoxylin-phloxin-safranin (HPS) staining methods. Stained sections were scanned with a Nanozoomer apparatus (Hamamatsu). To assess batch quality, histological analyses were conducted on a subset of SEs (*n*=15) from most batches. SEs were evaluated based on four criteria and assigned a histological score out of 10, with 10 representing the highest quality. Only batches with a mean score of 6 or higher were included in the study. A set of sections, infected and non-infected, was observed by a skilled pathologist to detect lesions, notably attributable to a herpesvirus infection.

### RNA extractions, reverse transcription and real-time quantitative PCR

SE fragments and leg skin used as control were snap frozen in liquid nitrogen and stored at −80 °C. SEs were lysed in the presence of 1.4 mm ceramic beads (#19-645-3 OMINFIT) by mechanical disruption using a Precellys® 24 Tissue-Homogenizer instrument (Bertin Technologies) with 300 µl RLT lysis buffer (#74104, Qiagen) containing 0.1% *β*-mercaptoethanol extemporaneously added. The lysate was then incubated for 30 min at 56 °C in the presence of proteinase K according to the manufacturer’s protocol adapted for skin tissue (Appendix C: RNeasy mini protocol for isolation of total RNA from heart, muscle and skin tissue, #74104, Qiagen). Tissue debris were removed by centrifugation, and RNAs were extracted from the collected supernatant by using the RNeasy minikit (#74104, Qiagen). RNA samples were then treated enzymatically with RNase-free RQ1 DNase (1 UI µg^−1^ RNA, #M6101, Promega kit), and RNA concentrations were measured with a NanoDrop spectrophotometer. Total RNAs were reverse transcribed into cDNA by using Moloney Murine Leukaemia virus (MMLV) reverse-transcriptase (#M1701, Promega) with 250 ng µg^−1^ RNA Oligo(dT), 200 UI µg^−1^ RNA of MMLV RT (#C110A, Promega) and dNTP (#U151A, Promega), all diluted in 1× MMLV buffer as instructed by the manufacturer’s protocol. Real-time quantitative PCR (qPCR) was performed in triplicate on a C1000 Touch CFX96 RealTime System (Bio-Rad, arnes-la-Coquette, France) with an iQ Supermix SYBR green probe (#1708882, Bio-Rad). Primers (synthesized by Eurogentec) specific for the gene of interest were utilized (Table 1). The qPCR programme consisted of one 5-min activation step at 95 °C followed by 40 cycles alternating 20 s at 95 °C and 35 s at 60 °C. The chicken ribosomal protein S17 gene (RPS17) served as a housekeeping gene reference. Each mRNA level of our genes of interest was normalized relatively to RPS17 expression by the 2^-∆Ct^ analysis method and was expressed in an arbitrary unit (A.U.).

### Dermis/epidermis separation from SE and corneocyte purification

The epidermis equivalent was separated from the dermis equivalent by incubating SE (collected at D14) with 2 mg ml^−1^ thermolysin (Sigma) at 4 °C for 3 h and used for subsequent analyses. To purify corneocytes, small cut epidermis pieces were then incubated in 1 ml extraction buffer (Tris-HCL pH 7.4 20 mM, EDTA 5 mM and SDS 2%] completed with DTT (10 mM final solution) before a 40-min agitation in an Eppendorf Thermomixer Comfort (set at 95 °C and 500 r.p.m.). This boiling step (using a buffer containing DTT) denatures proteins. Debris were let settled, and the supernatant was transferred in another 1.5-ml tube to be centrifuged at 13,000 r.p.m. for 10 min. The pellet was washed two times with a washing buffer (Tris-HCL pH 7.4 20 mM, EDTA 5 mM and SDS 0.2%) with DTT (10 mM final solution). Finally, the pellet was resuspended in H_2_O. Purified corneocytes were plated onto 0.17-μm-thick glass coverslips by centrifugation at low speed (500 ***g***, 800 r.p.m.) for 5 min using a Cytospin 4 centrifuge (Thermo Fisher Scientific). Nuclei were counterstained with Hoechst 33342 dye (1:2,000 dilution; Invitrogen), and the coverslips were mounted with Mowiol mounting medium (Merck). Images were obtained on an Axiovert 200 M inverted microscope (Zeiss) using a 63× Plan-Apochromat objective (NA=1.4, Zeiss), mounted with a Moment CMOS camera (Teledyne Photometrics) and driven by Inscoper hardware and software (v8.5.10). Images were processed using ImageJ software (1.54j).

### DNA extraction from SE and corneocytes

Snapped frozen SE or purified corneocytes (as described above) were mechanically disrupted into 360 µl ATL lysis buffer (QIAamp DNA Mini Kit, Qiagen) using a Precellys® 24 Tissue-Homogenizer instrument as previously described for RNAs. Forty microlitres of proteinase K were added to the samples before incubating it overnight at 56 °C and extracted with the QIAamp DNA Mini kit (Qiagen), according to the manufacturer’s protocol, except that twice washing buffer was used. DNA concentrations were determined with a NanoDrop spectrophotometer.

### Absolute quantification of viral genomes by TaqMan qPCR

Viral genome copies were quantified through real-time qPCR using TaqMan technology as described previously for MDV [40] and HVT [26]. In brief, MDV genome was quantified with ICP4 gene, HVT genome with non-coding sequence and cellular genome with chicken iNOS gene. All qPCRs (except for corneocytes, see below) were performed independently in triplicate, for the viral gene and for iNos, with 250 ng DNA from SEs, 10 pmol of each gene-specific primer and 5 pmol of the gene-specific probe in a 20 µl volume on a CFX96TM Real-Time C1000 Touch™ Thermal Cycler (Bio-Rad). The results were analysed using CFX Manager software (v3.1) (Bio-Rad). For each sample, the MDV or HVT genome copy number was reported relative to one million cells. MDV genomes were also quantified in purified corneocytes. For that, only ICP4 qPCR reactions were performed, because cellular DNA is degraded during corneocyte differentiation. Because a low amount of DNA was extracted from this material, each qPCR on corneocytes was performed with 20 to 45 ng of DNA.

### SE cryosections, staining and fluorescence microscopy

SEs were snap frozen in nitrogen-cooled isopentane. Ten-micrometre-thick cryosections were performed using a Leica CM 3050 S Cryotome (Leica), fixed in 4% paraformaldehyde for 15 min, washed three times in PBS and then permeabilized/saturated 30 min at room temperature with a 2% BSA and 0.1% Triton X-100 PBS solution. Some sections were then stained overnight at 4 °C with a mouse monoclonal antibody anti-Keratin (KRT) (11E10) that recognizes several type I KRTs, mainly KRT3, 12, 14 and 18 (kind gift of Danielle Dhouailly [41]) at 1:400, in 1% BSA and 0.1% Triton X-100 PBS solution. The next day, sections were washed three times with PBS and incubated for 45 min at room temperature with secondary Alexa Fluor 555-conjugated goat anti-mouse antibody (Invitrogen) diluted in 1% BSA and 0.1% Triton X-100 PBS solution. At the end, for all sections stained with 11E10 or not, nuclei were counterstained with Hoechst 33342 (1:2,000 in PBS) (Invitrogen), washed three times in PBS before being mounted on slides using a Mowiol/DABCO mixture. Samples were observed on an Axiovert 200 M inverted microscope (Zeiss) using a 63× Plan-Apochromat objective (NA=1.4, Zeiss), mounted with a Moment CMOS camera (Teledyne Photometrics) and driven by Inscoper hardware and software (v8.5.10). Some sections were also observed on an AX confocal microscope system (Nikon). All images were processed using ImageJ software (1.54j).

### Transmission electron microscopy

Small pieces of infected SE were cut with a scalpel blade under a stereomicroscope and fixed in 1% glutaraldehyde and 4% paraformaldehyde (Sigma) in 0.1 M phosphate buffer (pH 7.2). Samples were prepared as previously described [42]. Ultrathin sections (50 to 80 nm) were performed with an EM UC7 ultramicrotome (Leica). Ultrathin sections were stained with 5% uranyl acetate (Agar Scientific) and 5% lead citrate (Sigma), and observations were made with a transmission electron microscope JEOL 1011 (Jeol). Images were acquired using a Rio9 camera (Gatan) and processed with Digital Micrograph v3.42.3048.0 software (Gatan).

The surface of infected SE, corresponding to the cornified layer, was scrapped with a scalpel blade under a fluorescent stereomicroscope MZ10F (Leica). This material was imaged using the stereomicroscope to verify the red fluorescence and next prepared for transmission electron microscopy (TEM) as above.

### Statistical analyses

All graphs and statistical analyses were performed using GraphPad Prism software v8 (GraphPad Inc). Data are presented as means and sd or median and interquartile range. When the Mann–Whitney test was used, an exact *P*-value <0.05 was considered statistically significant. The Kruskal–Wallis test with a Dunn correction for multiple comparison was used. Adjusted *P*-value <0.05 was considered statistically significant as indicated in figure legends.

## Results

### Validation of chicken SE reconstruction using embryonic primary chicken keratinocytes

We first aimed at reconstructing SEs from primary chicken keratinocytes from embryos (CPKe) instead of 1-day-old chicks as reported previously [5]. As before, CPKe were isolated from the leg skin. In addition, we assayed SE reconstruction, either exclusively with CPKe or by adding K8 cells. K8 is a chicken keratinocyte line derived from chicken embryonic stem cells [34], which was previously shown to be permissive to MDV replication [35]. In brief, SE reconstruction was performed as follows (Fig. 1a): in an insert, a dermis equivalent was reconstituted on the top of which basal keratinocytes (CPKe±K8 cells) were seeded. First, SEs were cultured for 2 days in medium and then at ALI from day 2 (D2) until day 14 (D14), the day at which the skin reconstruction was stopped. At D14, SEs with or without K8 displayed the expected morphological characteristics of reconstructed chicken skin *in vitro* as observed by white light microscopy after transversal sectioning and HPS staining (Fig. 1b). At low magnification, the epidermis appeared well stratified, displaying its four distinct layers (Fig. 1b). Compared with natural chicken skin, some differences were noted as earlier [5], including a thicker epidermis, particularly in the innermost layer (basal) and the outermost eosinophilic cornified layer. Additionally, some vacuolization of keratinocytes was observed and some variability between batches of SEs.

To validate this SE model, we next assessed the RNA expression of four specific epidermal markers using Reverse Transcriptase-quantitative Polymerase Chain Reaction (RT-qPCR) in comparison with 1-day-old leg skin (Fig. 1c). Keratin 14 (KRT14), a marker of the undifferentiated state of keratinocytes, was expressed in both types of SE (4 to 8 A.U. median). In SE, this marker was more expressed than in normal skin (1 A.U.), as found earlier [5] and consistent with the thickness of the basal layer, although the difference was not significant. The transcripts of three proteins that are naturally abundant in suprabasal layers, KRT9-L3 (a type I keratin), KRT78L3 (a type II keratin) and loricrin 1 (LOR1, a protein from the epidermal differentiation complex), were next examined. In both SEs, the transcripts encoding these three proteins were expressed at similar levels compared with chicken skin (Fig. 1c).

Immunofluorescence analysis with 11E10 monoclonal antibody revealed that KRT14, possibly together with closely related other type I keratins, is present in the basal layer and disappears as keratinocytes differentiate in the upper layers as expected (Fig. 1d). In addition, DNA staining with Hoechst confirmed the presence of nuclei in the basal layer and suprabasal layer and their near absence in the cornified layer as expected (Fig. 1d).

These results indicate that markers of skin differentiation of the suprabasal layer are expressed comparably between SEs and chicken skin legs and that KRT14 is present in undifferentiated keratinocytes of the lower layers. Altogether, these findings reveal that CPKe from chicken embryos, with or without added K8 cells, effectively supports the reconstruction of 3D chicken skin.

### Chicken SE is susceptible to MDV infection and MDV infection does not impede SE reconstruction

**Fig. 2.**
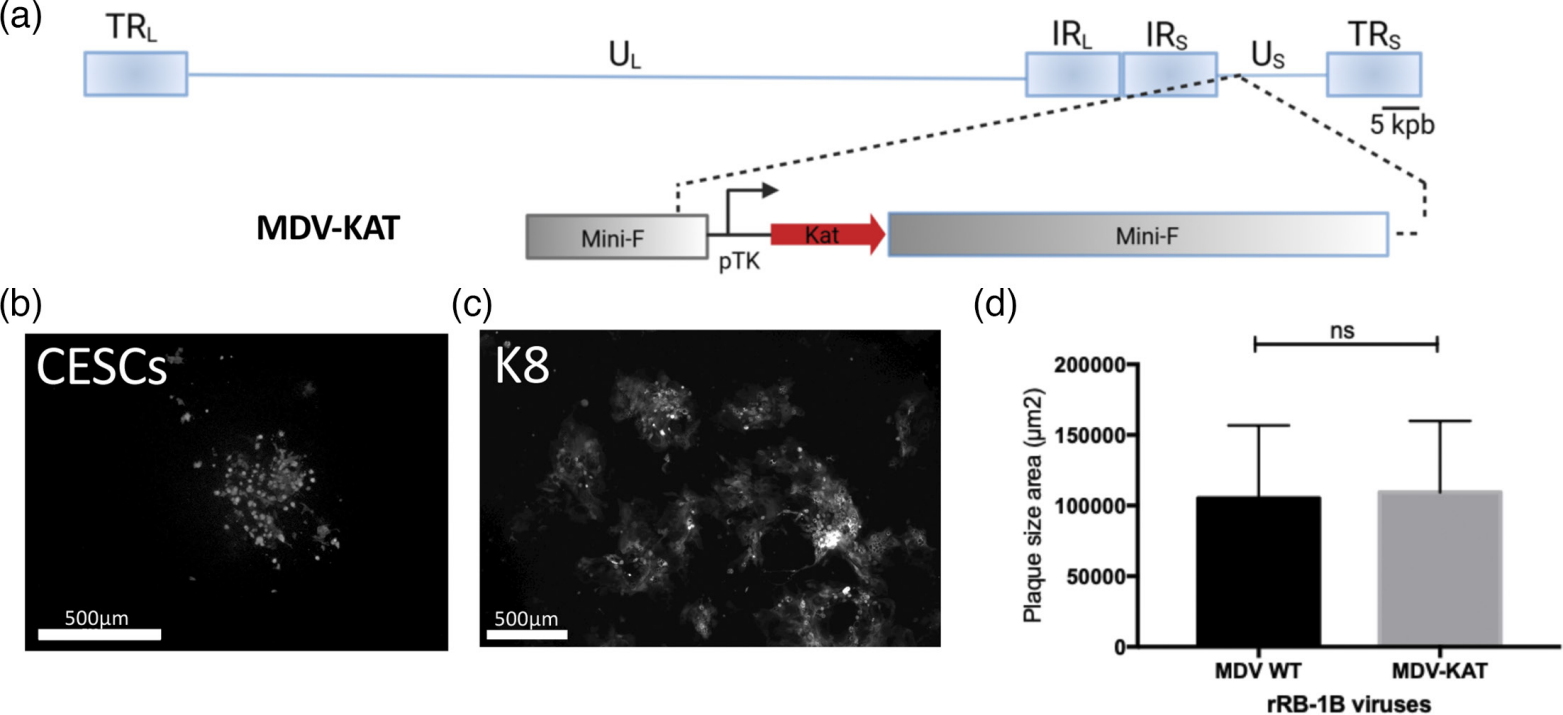
Generation and characterization of the MDV-KAT virus *in vitro*. (**a**) Overview of the MDV RB-1B genome with a focus on the mini-F cassette with the Katushka fluorescent reporter gene under the TK promotor (MDV-KAT), shown as a red arrow. Image of MDV-KAT-infected CESCs (**b**) and MDV-KAT-K8 cells (**c**) showing bright red fluorescent plaques. Scales bars, 500 µm. (**d**) Plaque size assays of the indicated viruses 4 days post-infection in CESCs. The plaque diameter is shown as bars with mean and sd. *P*>0.5 (ns, non-significant), Mann–Whitney test (*n*=50).

Given that SEs exhibit high auto-fluorescence in the green channel, we developed a red fluorescent recombinant MDV utilizing the BAC system, based on the very virulent RB-1B strain. This mutant, designated MDV-KAT, expresses the Katushka fluorescent protein (KAT) under the control of the early HSV-1 TK promoter (Fig. 2a). Infection of CESCs with MDV-KAT resulted in the formation of bright red fluorescent plaques in both CESCs (Fig. 2b) and K8 cells (Fig. 2c). Plaque size assay showed that MDV-KAT spreads in CESC culture *in vitro* comparably to the WT rRB-1B virus (Fig. 2d), indicating that the Katushka fluorescent gene does not impair MDV propagation in cell culture.

**Fig. 3.**
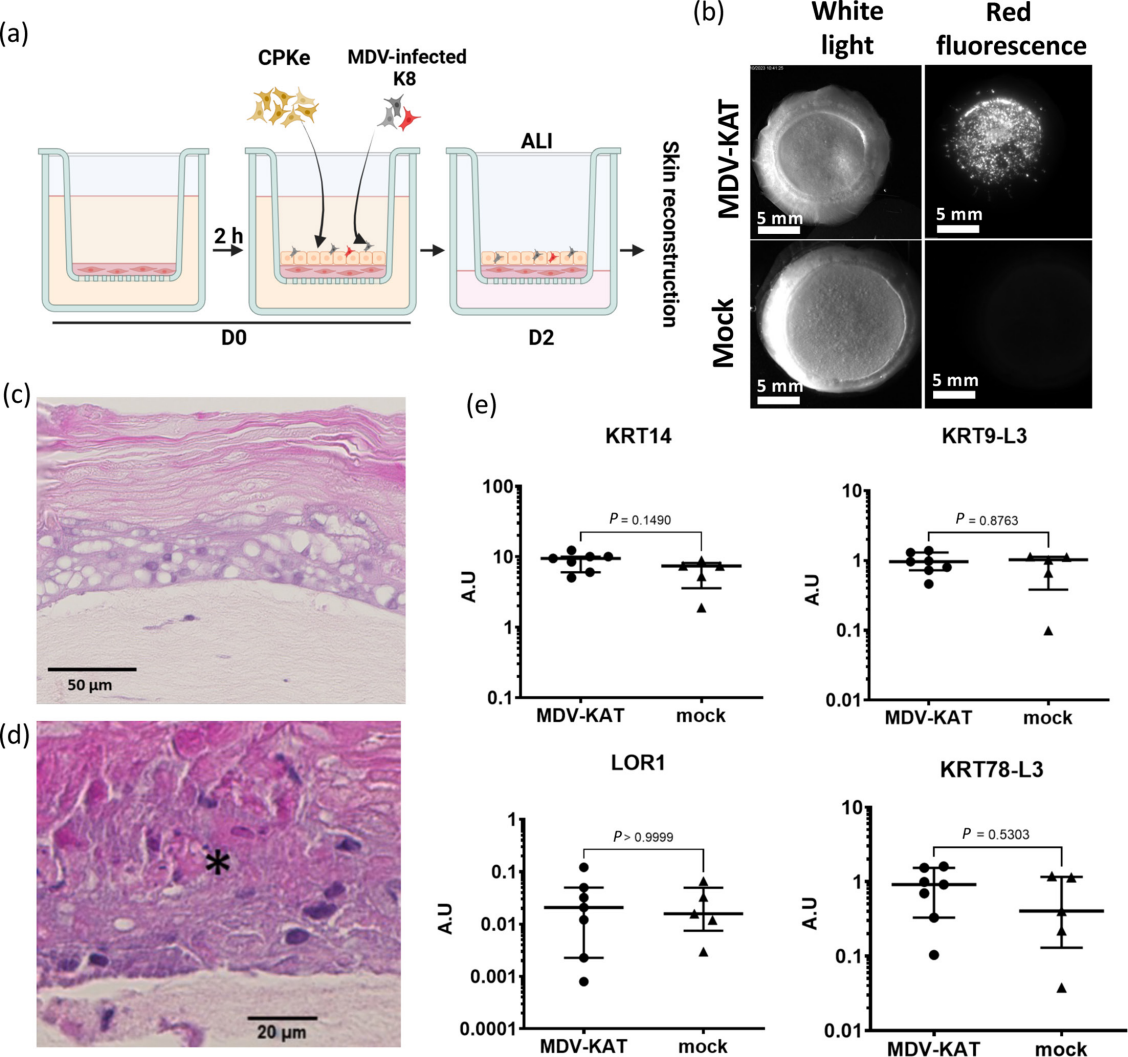
MDV efficiently infects and replicates in SE. (**a**) Schematic overview of the SE infection with MDV. (**b**) Morphological macroscopic aspect under white light of SE infected with the very virulent red fluorescent MDV-KAT or mock-infected SE (with K8) at D14 post-seeding and infection (scale bar, 6 mm; 250-ms exposure time). Red fluorescent imaging of MDV-KAT and mock-infected SE using a fluorescence stereomicroscope to evaluate MDV infection (scale bar, 6 mm; 250-ms exposure time). (**c, d**) Histological analysis included H- and E-stained sections of MDV-KAT-infected SE at two magnifications. In (**d**), a lesion is highlighted by an asterisk. (**e**) The mRNA expression levels of skin differentiation markers in MDV-KAT and mock-infected SE were quantified. Each symbol corresponds to one SE, performed on four independent experiments (MDV-KAT, *n*=7; mock, *n*=3) presented here (for the mock, same data shown in Fig. 1). mRNA levels for each gene were normalized to the housekeeping gene RPS17, were expressed in A.U. and were presented in a dot plot showing median values±interquartile range.

We next investigated whether the SE model is permissive to MDV-KAT infection and assessed the impact of infection on SE reconstruction. Currently, MDV infection *in vitro* can only be achieved by co-cultivating uninfected cells with MDV-infected cells. Therefore, we seeded the dermis equivalent with CPKe and K8 cells infected with MDV-KAT (Fig. 3a) or mock infected (Fig. 1a). We then followed the SE reconstruction protocol as outlined above. Importantly, it should be noted that the inoculum cannot be removed in this infection protocol. At D14, the infected SEs displayed macroscopic morphology similar to that of the mock-infected SEs (Fig. 3b, left panel). Imaged using a fluorescent stereomicroscope, the MDV-KAT-infected SE showed intense red fluorescence, in contrast to the mock-infected control, which showed no signal (Fig. 3b, right panel). The fluorescent Katushka signal appeared as numerous infection plaques distributed across the entire SE surface. This observation was consistent across 22 MDV-KAT-infected SEs produced in 9 independent experiments, although fluorescence intensity varied slightly between experiments.

Microscopic analysis of HPS-stained sections from infected SEs revealed full SE reconstruction (Fig. 3c) as mock infected (Fig. 1c), indicating that MDV infection did not interfere with 3D skin reconstruction. A comprehensive comparison between MDV- and mock-infected SEs was conducted to identify any lesions induced by viral infection. Lesions observed exclusively in the MDV-infected SE were rare, primarily consisting of few and small clusters of necrotic keratinocytes with fragmented, hyper-eosinophilic cytoplasm and dark, shrunken morphology (Fig. 3d).

To further confirm that SE reconstruction remained essentially unaffected under infection conditions, we assessed the expression of skin differentiation markers in the infected SEs compared with the mock-infected controls. Expression levels of the four markers previously evaluated (KRT14, KRT9-L3, KRT78-L3 and LOR1) were measured and found to be not significantly different between the two conditions (Fig. 3e), supporting macroscopic and microscopic observations.

In summary, our results demonstrate that MDV successfully infects SEs, resulting in numerous infection plaques. Importantly, based on histology and expression of keratinocyte markers, MDV infection does not compromise the SE reconstruction.

### MDV primarily replicates and spreads during the first week of SE reconstruction, with viral persistence sustained thereafter

**Fig. 4.**
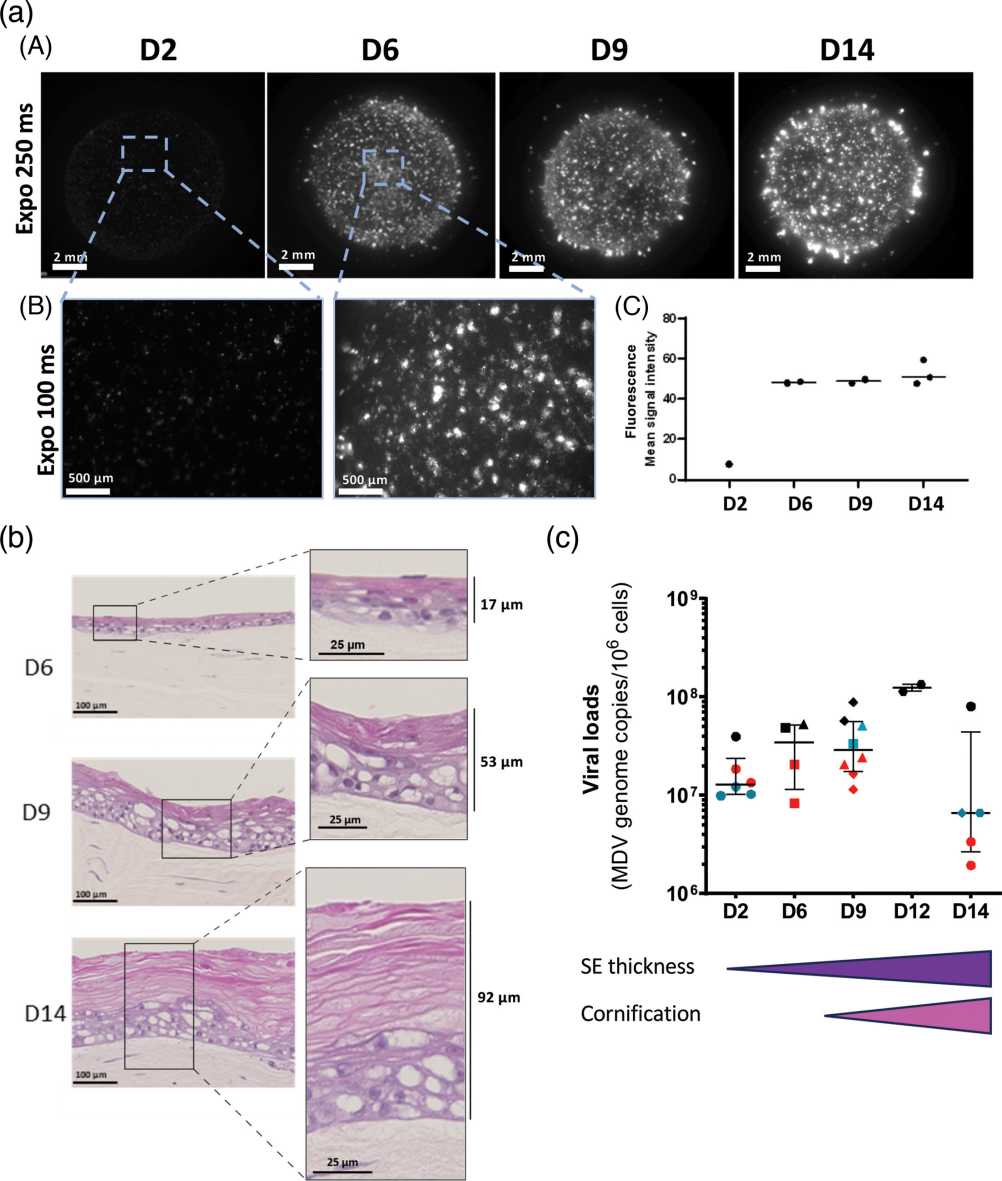
MDV infection in SE followed by fluorescence imaging and viral genome loads at different timepoints. SEs were infected at D0 with MDV-KAT, and viral infection was assessed into SEs throughout time, during reconstruction (D2, D6, D9 and D14 post-seeding time). (**a**) SEs were imaged using a fluorescent stereomicroscope. (A) The entire SEs were imaged using the same exposure time. (B) Portions of SE were imaged at higher magnification (×80). (C) Quantification of the red fluorescent signal on independent SEs (*n*=1 to 3) from a single kinetic experiment. (**b**) Histological structure of MDV-infected SEs at indicated timepoints. Formalin-fixed SEs embedded in paraffin were sectioned and stained in HPS. The number of layers in the epidermis increased over time as expected and in consequence its thickness (indicated on the side of enlarged panels). The corneum layer started to appear at D9 and was highly developed at D14. (**c**) Viral genome loads of infected SE were determined at different timepoints. DNA was extracted from SE and viral genomes were quantified by real-time qPCR, and their numbers were indicated per million nucleated cells. The cell number determination is based on the detection of iNos cellular DNA sequence. Results are presented in a dot plot showing median values±interquartile range. To show inter- and intra-experiment variations between SEs, each independent experiment was represented in a different colour (three independent assays are shown) and each SE with a symbol. Two similar symbols of the same colour mean quantification from two parts of the same SE.

In order to further characterize MDV infection of SEs, kinetics of infection were followed by fluorescence imaging through a stereomicroscope (Fig. 4a), histological examination (Fig. 4b) and viral load quantification (Fig. 4c) at different timepoints post-infection (D2, D6, D9, D12 and/or D14). Stereomicroscopy reveals that red fluorescence intensity increased highly between D2 and D6 and then remained constant up to D14 (Fig. 4a, panel A). At D2, the signal was slightly visible and comes in the form of tiny dots (Fig. 4a, panel B). In contrast, in the same conditions, at D6, the signal was nicely visible as dots of larger size and irregular shape, compatible with MDV infection plaques (Fig. 4a, panel B). From D6 to D14, the red fluorescence appeared of similar intensity and shape or size (Fig. 4a, panel A). This was confirmed by quantifying the fluorescence intensity over time (Fig. 4a, panel C).

In order to better understand MDV infection in relation to the stages of SE reconstruction, we analysed the histological structure of MDV-KAT-infected SE at three time points (D6, D9 and D14). This revealed that at D6, the SE exhibited a thickness of about 17 µm, with only four to five layers of cells, including a thin intermediate layer. By D9, SE stratification progressed with the appearance of the transitional layer and of a thin cornified layer (epidermis thickness of about 50 µm). In addition, between D6 and D9, the intermediate layer became thicker. Between D9 and D14, we observed mostly the development of the stratum corneum, whose thickness increased considerably, leading to an epidermis thickness of about 90 µm (Fig. 4b).

We next measured virus loads in SEs over time. To achieve this, DNA was extracted from a sample of SE and MDV genome copy number per million cells were determined by absolute qPCR. This method allows the quantification of all viral DNA and nuclear DNA in the course of replication and incorporation into viral particles. Viral loads were already high at D2 (median value of 1.8×10^7^) (Fig. 4c), which is coherent with the input of 500,000 MDV-infected-K8 mixed of CPKe at D0, and the beginning of viral replication. From D2 to D9, viral load medians moderately increased (median value of 3.5×10^7^ at D6 and 3×10^7^ at D9). Statistical analysis was not feasible herein due to a mixture of paired and unpaired data. Of note, the number of cells by surface unit increases over time, as the SE grows. In addition, corneocytes have lost their nuclei and therefore cannot be quantified by qPCR, leading to an underestimate of viral loads and cell numbers. At D14, although inter-assay variations were higher than at earlier time points, viral loads decreased in all assays compared with D9 (Fig. 4c). This result is probably a consequence of the normalization method we used to calculate viral loads and of the cornification process, which will be discussed later.

In summary, regarding the assessed conditions and time points, the MDV infection of the SE, as evidenced by fluorescence and viral loads, progresses from D2 to D6, coinciding with the formation of the intermediate layer. From D6 to D9, SE stratification still continued, whereas MDV infection remained constant. From D9, as SE reconstruction pursued, mostly by cornification, MDV infection was sustained as shown by fluorescence but reduced according to virus loads.

### MDV infection is located in the upper layers of fully reconstructed SEs, including in corneocytes

**Fig. 5.**
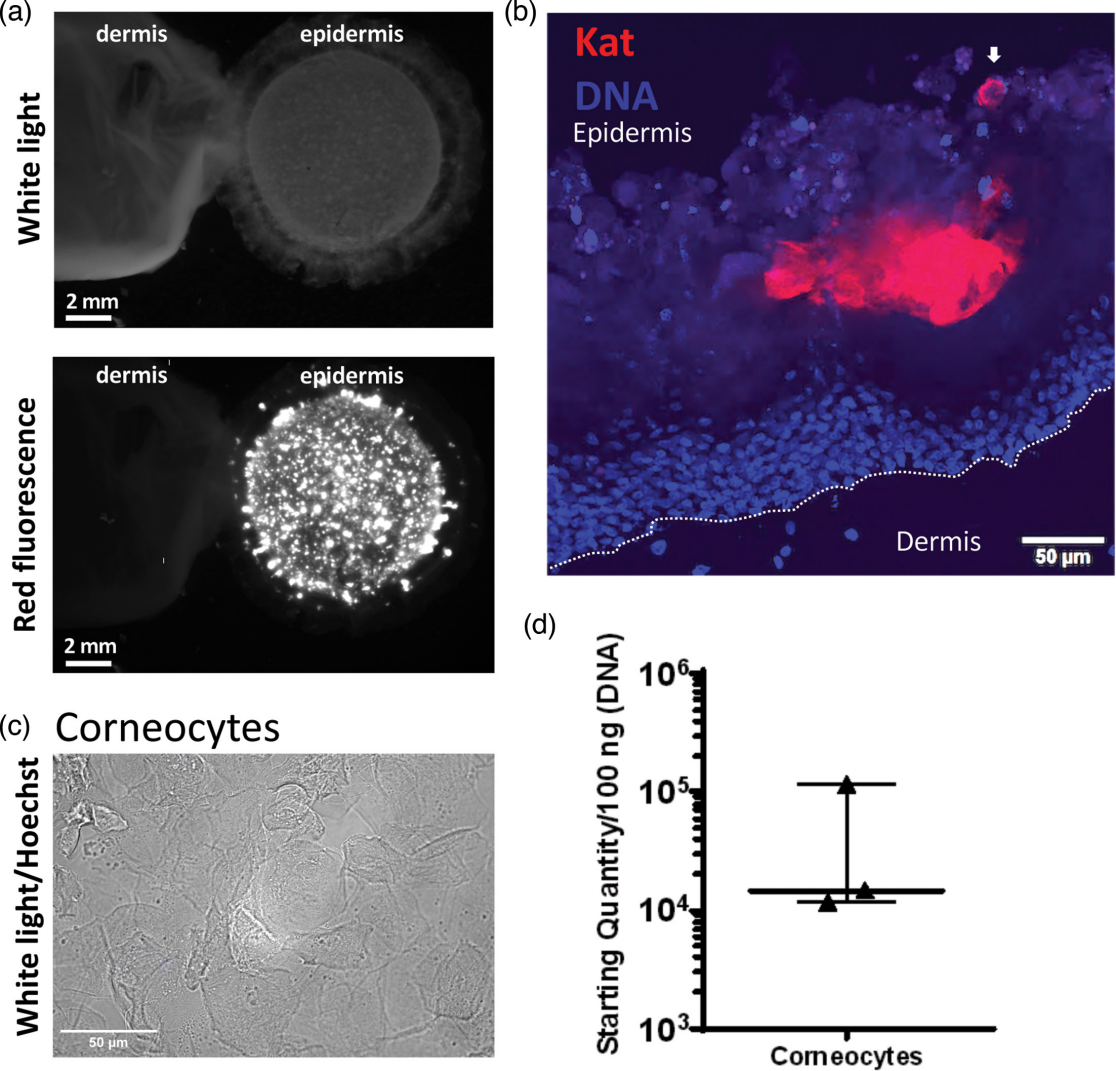
MDV is located in the upper layers of the reconstructed epidermis, notably in corneocytes. (**a**) MDV-KAT-infected SE (at D14) was treated with thermolysin, and the reconstructed epidermis was separated from the dermal equivalent. Both the dermis and epidermis were imaged using a fluorescent stereomicroscope under white light (upper panel) and fluorescence (lower panel). The epidermis showed numerous fluorescent infection plaques, but no fluorescence signal was observed in the dermis. (**b**) Cryosections of MDV-KAT-infected SEs were stained with Hoechst and directly observed by confocal microscopy. The red fluorescent signal indicates that the infection was localized in the suprabasal layers of the epidermis. The white dashed line indicates the boundary between the epidermis and dermis. The white arrows pointed to red fluorescent cells with a typical morphology of corneocytes. (**c**) Corneocytes were purified from the entire reconstructed epidermis infected with MDV-KAT, stained with Hoechst 33342 dye and subsequently examined by light and fluorescence microscopy. The cells had the shape of corneocytes with no nuclei as expected. (**d**) Viral genome loads in corneocytes. DNA was extracted from purified corneocytes, and viral loads per 100 ng of total DNA were quantified by RT-qPCR using the ICP4 gene. No normalization relative to iNos copy number was performed due to cellular DNA degradation during final keratinocyte differentiation into corneocytes.

To localize the site of MDV-KAT infection in fully reconstructed SE at D14, the epidermis was separated from the dermis and both parts were observed under a fluorescent stereomicroscope. No fluorescence was detected on the dermis part, whereas fluorescent plaques were visible on the epidermis part, like on the full SE (Fig. 5a). This result indicates that MDV infection remained in the epidermis and did not spread to the dermal compartment. To determine in which layers of the epidermis MDV infection was located at D14, areas containing a strong fluorescent signal of an MDV-KAT-infected SE were sampled and frozen. After DNA staining with Hoechst, cryosections were observed by confocal microscopy. Red foci, indicative of infection, were detected mostly in the intermediate layers and some corneocytes (Fig. 5b).

To determine if red fluorescence in the cornified layer was associated with the presence of viral material, we first purified corneocytes according to a specific procedure based on the high physical resistance of these ‘dead’ keratinocytes mostly consisting of cross-linked proteins. Purified cells observed on a light microscope had corneocyte morphology. These cells had no nuclei detected through Hoechst staining, as expected (Fig. 5c). Next, DNA was extracted from these purified cells. The quantity of DNA obtained from these cells was very low (between 100 and 650 ng cm^2^ per piece of epidermis, on three independent experiments) in accordance with the expected degradation of nuclei during cornification. By qPCR, the viral MDV genome was detected with a starting quantity of 1.5×10^4^ per 100 ng of DNA (Fig. 5d), whereas the iNos gene reflecting cellular DNA was almost undetectable (Ct >35). This shows that viral DNA is detectable in corneocytes purified from SE epidermis, despite the loss of nuclei, suggesting that some viral DNA was present and protected from DNA degradation during the cornification process.

Altogether, these results indicate that MDV infection is present in the intermediate and the cornified layer of the epidermis of SEs, but not in the dermis.

### Validation of the presence of virions in the upper layers, including in the cornified layer, by electron microscopy

**Fig. 6.**
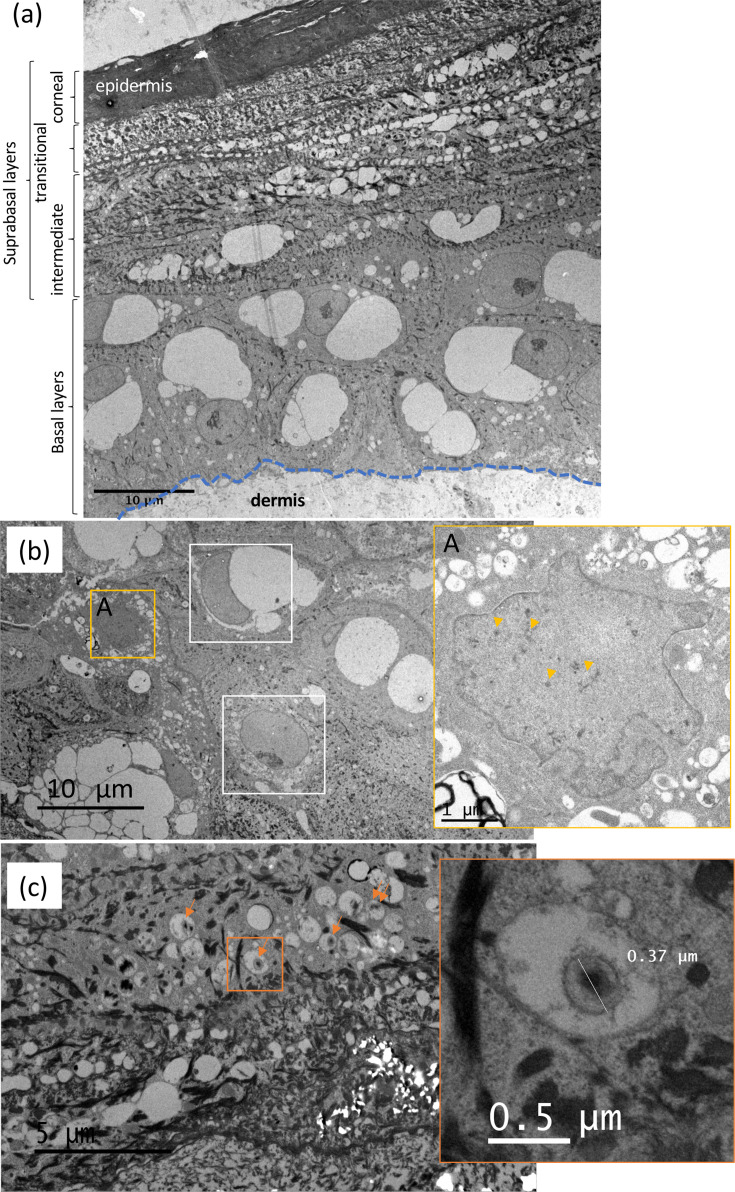
Viral particles are observed in the inner layers of the epidermis by TEM. MDV-KAT-infected SEs at D14 were collected and subsequently analysed at ultrastructural level (**a**). Image of the epidermis. The four layers with keratinocytes at different stages of differentiation are visible. Note that the cornified layer is highly electron dense. (**b**) Image of a zone with three keratinocytes (in framed), inside which viral particles were observed. All viral particles were nuclear capsids, of the three types (**a, b, c**). An enlargement of the cell framed in yellow is shown on the right panel. Yellow triangles indicate capsids. (**c**). Image of a zone where complete viral particles, indicated by orange arrows, are visible inside vesicles. An enlargement of a complete particle is shown on the right panel.

To ascertain viral infection in the upper layers of the epidermis and identify what kind of viral particles are present, ultrathin sections of MDV-KAT-infected SE were examined by TEM. At low magnification, the four layers of the epidermis were visualized (Fig. 6a), supporting what was observed on HPS-stained sections (Fig. 3b). The cornified layer was highly electron dense and compact. MDV particles were detected in the inner layers of the epidermis. Specifically, we observed particles in keratinocytes of the second or third layer of cells, at the interface between basal and intermediate layers, corresponding to large cells with a large nucleus. Viral particles were visible only in the nuclei, with a low number of viral particles per infected cell (Fig. 6b). Intranuclear virions were naked capsids of the three types (A, B and C) (Fig. 6b, enlarged panel A). We also occasionally detected structures reminiscent of enveloped mature virions inside a cytoplasmic vacuole of flat keratinocytes in suprabasal layers (Fig. 6c). These structures had a diameter of 340–370 nm, a size compatible with mature MDV, although in the upper range (Fig. 6c, enlargement). No viral particles were observed inside cytoplasmic inclusions of electron-dense material, as reported by pioneer works in FFE [17,43]. Finally, although we did not observe herpesvirus particles in the cornified layer, we cannot rule out the possibility that they were missed due to the high electron density and compactness of this region.

**Fig. 7.**
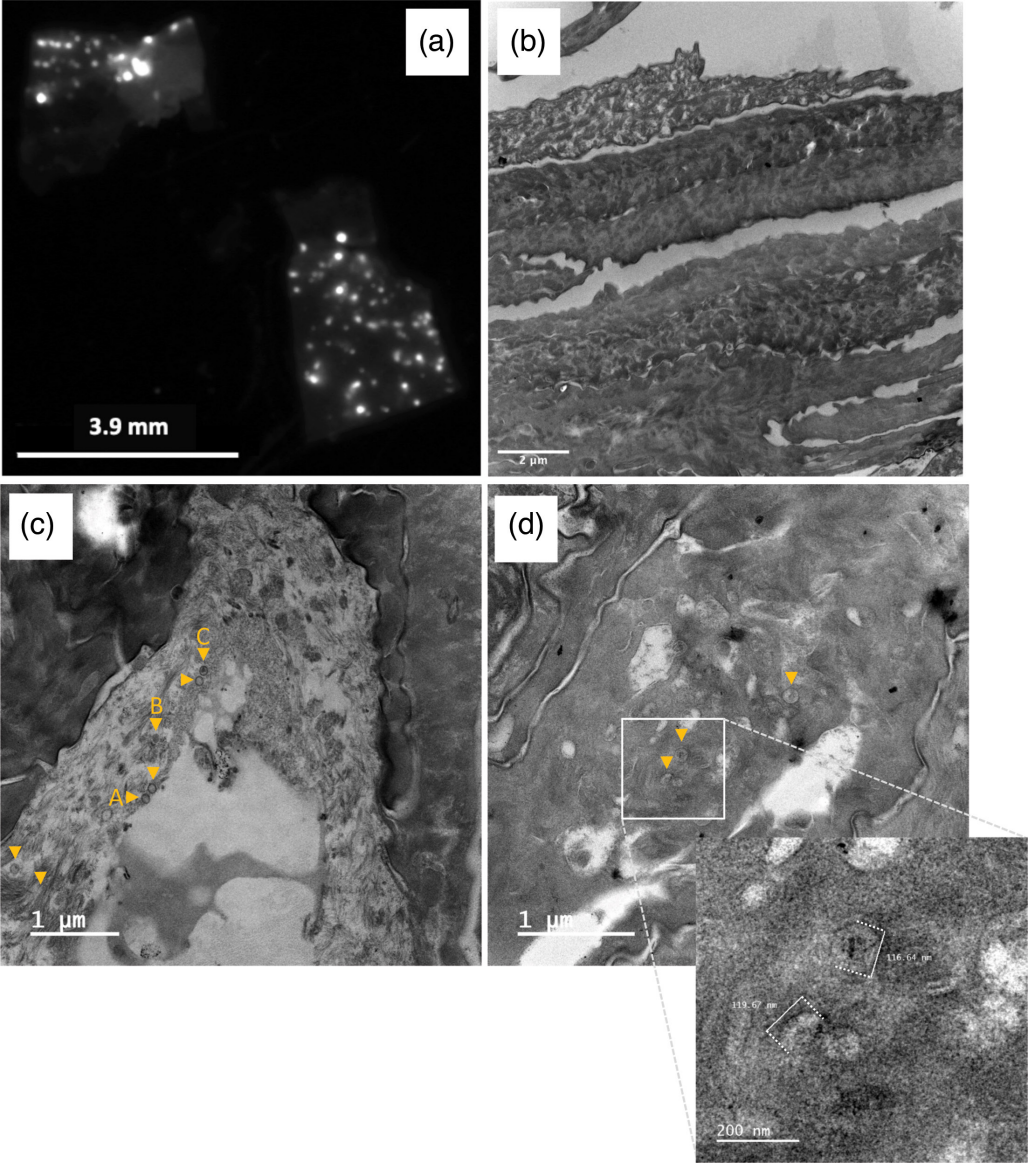
Examination of the cornified layer by fluorescence stereomicroscopy and TEM. The cornified layer was harvested on fresh MDV-KAT-infected SE at D14 and subsequently analysed. (**a**) Image of the freshly harvested material under a fluorescent microscope on the red channel. Numerous small MDV plaques are visible. (**b**) Examination of the harvested material at ultrastructural level by TEM. The material has the aspect of several layers of cornified cells as expected for the cornified layer. The cells were highly electron dense as observed earlier in the cornified layer on entire SEs. (**c, d**) Images of corneocytes at ultrastructural level, in which viral particles were observed. Herpesvirus capsids (A, B, C) are visibly surrounded by cellular fibres. No nucleus was visible. The yellow triangles indicated particles.

To examine more carefully the cornified layer for the presence of virions, as suggested by the finding of viral DNA in purified corneocytes (Fig. 5d), we next harvested the cornified layer by scraping it on fresh MDV-KAT infected SE at D14. Examination of the scraped material by fluorescence using a stereomicroscope confirmed the presence of infected cells (Fig. 7a). Ultrathin sections of this material were subsequently examined by TEM and showed typical morphology of the stratum corneum, with rows of enucleated flattened corneocytes, highly electron dense (Fig. 7b). At higher magnification, capsids were detected in a few cells of lower electron density (Fig. 7c) and cells of high electron density (Fig. 7d). All these structures appeared as naked capsids, with a diameter of 116–120 nm (Fig. 7d, enlarged panel), whereas no mature virions were detected in these cells.

### MD vaccine viruses can also infect and replicate in SE

**Fig. 8.**
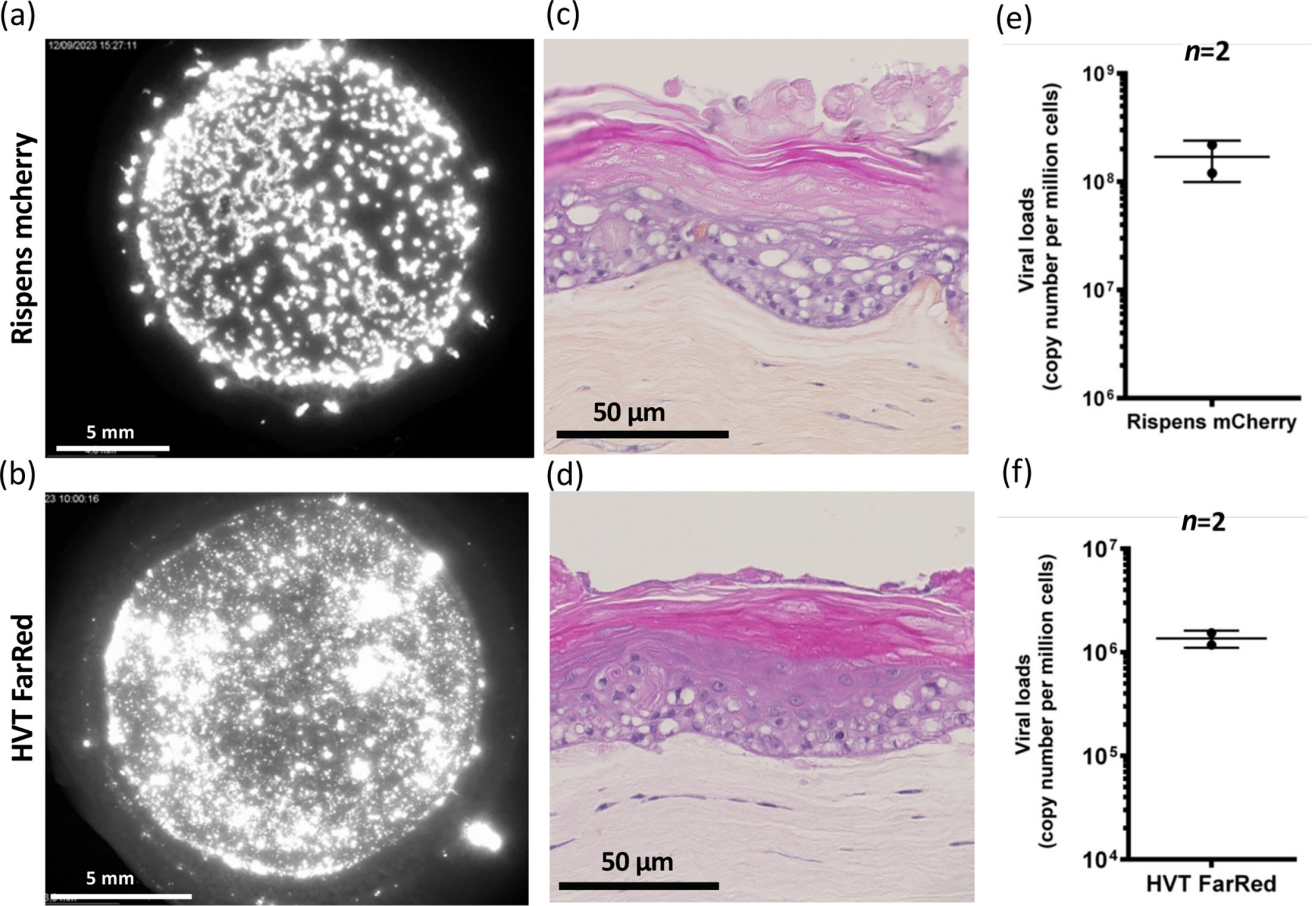
SEs are infectable by vaccinal MD strains, Rispens and HVT. SEs were produced by mixing CPKe with K8 cells infected with recombinant fluorescent vaccinal strains, Rispens mCherry or HVT FarRed, as performed for MDV. Analyses were performed on reconstructed SEs at day 14. (**a, b**) Red fluorescence images of infected SEs were performed using a fluorescent stereomicroscope to detect infection, with Rispens mCherry (**a**) or HVT FarRed (**b**). (**c, d**) HPS stained sections for both infected SEs, with Rispens mCherry (**c**) or HVT FarRed SE (**d**). (**e, f**) Viral load measurement (viral genome copies/10^6^ cells) in SEs, with Rispens mCherry (**e**) or HVT FarRed (**f**).

To study if our 3D skin model can be infected with other GaAHV-2 strains and related mardivirus species, we performed SE infection with CVI988/Rispens and HVT, two non-pathogenic viruses used as MD vaccine and/or vaccine vector. For that, we used recombinant viruses expressing a red fluorescent protein, the Rispens mCherry and the HVT FarRed. SEs were infected with a protocol similar to the one used for MDV-KAT and analysed at D14 post-infection. Both viruses induced numerous fluorescent plaques of infection, although with different aspects: Rispens mCherry plaques resembled those of pathogenic MDV (Fig. 8a), whereas HVT FarRed plaques consisted of a multitude of small plaques in addition to some very large ones (Fig. 8b). Microscopic examination of HPS sections obtained after SE infection with these vaccine viruses showed that SE reconstruction appeared complete (Fig. 8c, d). Viral loads measured on two independent SEs at D14 were 1.69×10^8^ copies/million cells for Rispens and 1.36×10^6^ copies/million cells for HVT. Altogether, these results indicate that Rispens and HVT can infect, replicate and spread in chicken SEs.

## Discussion

In this study, we present, for the first time, the successful infection and replication of an organotypic 3D chicken skin model with a virus. We took MDV and MD vaccines as model viruses, these viruses having a well-known interaction with chicken skin [1,15, 20]. Indeed, it is well established that MDV induces a robust lytic infection of the keratinocytes from the feather follicle, leading to efficient virion production in the intermediate layer and MDV shedding into the environment.

One very interesting and major result of our study is that MDV infection of the basal layer during keratinocyte seeding does not compromise the skin epithelium reconstruction. This characteristic was first demonstrated by macroscopic and microscopic morphology 14 days post-reconstruction. Importantly, the cornified layer appeared as developed in MDV-infected SE as in non-infected one. Accordingly, we observed little pathological lesions on the epidermis by histology and TEM that could be attributed to virus infection, unlike what was observed with other viruses in the 3D skin model [10]. The non-disruption of SE reconstruction by MDV infection was further confirmed by the RNA expression levels of three suprabasal keratinocyte differentiation markers. Indeed, MDV infection of chicken SEs did not significantly reduce the expression levels of KRT9L3, KRT78L3 and LOR1, which are the functional equivalents of KRT10, KRT1 and loricrin, respectively, in chicken skin [44,45]. In this regard, MDV appears different from two viruses having skin tropism, varicella zoster virus (VZV), a human alphaherpesvirus and orf virus, a zoonotic poxvirus. Indeed, it was shown that VZV infection downregulates KRT10 RNA expression, a suprabasal keratinocyte marker, in different *in vitro* models of human keratinocyte differentiation [9,46]. Similarly, orf virus infection of the human 3D skin model also induced a downregulation of KRT10 transcription, as well as KRT1 and loricrin, 8 or 10 days post-infection [10]. In addition, SE reconstructed normally after MDV infection, indicating indirectly that MDV infection does not alter keratinocyte proliferation. These results are in accordance with the fact that *in vivo*, MDV infection of the FFE does not impair feather morphogenesis and growth, in contrast to other bird viruses, such as the reticuloendotheliosis retrovirus (reviewed in [1]). Therefore, from our results, we can conclude that MDV does not alter keratinocyte stratification and differentiation in the chicken SE model. Nevertheless, further dedicated studies will be needed to examine more precisely the interplay between MDV and keratinocyte differentiation.

At day 14 post-infection (D14), infection of SE was detected as fluorescent plaques. MDV is a cell-associated virus known to spread exclusively from cell to neighbouring cell in fibroblasts or keratinocyte monolayers, with no extracellular virions detectable [30,31, 35]. The presence of fluorescent plaques in SEs suggests that MDV spreads from keratinocytes to neighbouring keratinocytes in this 3D model, as it does in monolayer cultures. However, in our study, it is not possible to determine whether MDV spreads from K8 to primary keratinocytes. In addition, we could not determine whether MDV spread only horizontally between cells within the same row and/or vertically between cells of different rows.

Kinetic analyses showed that fluorescence was readily detected in MDV-infected SEs from D6 of infection in the form of plaques, whereas it was very faint at D2 and visible only as tiny dots. Between D2 and D6, fluorescence gained in intensity and the plaque size grew. Thereafter, fluorescence intensity remained stable until D14. This indicates that MDV infection has spread at the earliest time of SE reconstruction, just after ALI lifting. This window in time corresponds to the beginning of the keratinocyte differentiation process associated with early epidermis stratification. Indeed, at D6, a microscopic examination of SE showed four to five rows of cells (Fig. 4B). In that period, keratinocyte differentiation is in its early stages; however, we cannot directly demonstrate the role of keratinocyte differentiation in favour of MDV replication and spread, as it was described for human papillomaviruses in human 3D skin models [47]. Indeed, we have shown earlier that MDV is able to replicate and spread in a monolayer of basal keratinocytes *in vitro* [35]. The stability of the fluorescence signal from D6 to D14 suggests that in this period, MDV infection does not spread anymore but persists. The fluorescence stability was also associated with stable viral loads, although an inflexion was regularly observed at D14. One possible interpretation of these results is that most keratinocytes were infected at an early time between D2 and D6 and survived lytic infection until the end of the experiment (D14). The apparent high resistance of chicken keratinocytes to MDV lytic cycle also observed in monolayer culture is surprising and deserves to be better deciphered.

At the ultrastructural level, we observed different types of viral particles in three localizations: (i) capsids in the nucleus of large cells at the boundary between basal and intermediate layers, (ii) mature enveloped virions in the cytoplasmic vacuole of flattened cells in the intermediate layer and (iii) capsids in cornified layers. Although SEs appeared well infected based on fluorescence imaging and viral loads measured by qPCR, the virions were not numerous, as observed *in vitro* with MDV [28,33, 35]. This contrasts with the FFE from infected chickens, where Calnek and Nazerian reported numerous virions, although no quantification was performed [17,43]. Various explanations can be proposed for this difference in virion production: the origin of keratinocytes (FFE versus leg skin) and/or the timing of infection. In the FFE, it is not possible to know for how long the epithelium was infected and if re-infection occurs regularly. Herein, SEs were infected for 14 days, and based on the results of cryosections and corneocyte purification, it is plausible that a large number of virions were in the upper layers at that time and not observed by TEM, because of the high electron density of this region (see below). In addition, unlike Calnek and Nazerian in FFE, we did not observe virions inside large electron-dense cytoplasmic inclusions [17,43]. The nature and the role of these structures in MDV morphogenesis or shedding being not known make it difficult to say if their absence in our model is critical or not.

One of the most exciting findings of our study is the detection of MDV infection in the outermost layer, corresponding to the cornified layer. First, we observed fluorescent cells exhibiting corneocyte morphology at the surface of the SE in cryosections of the infected SE. The fluorescence in the cornified layer in this model is in coherence with the fluorescence detected in the outer sheath of feathers plucked from chicken infected with a fluorescent MDV [48], also corresponding to a cornified layer. Secondly, we detected viral DNA in corneocytes purified after boiling. It is interesting to point out here that very little DNA was extracted from purified corneocytes, as expected in these cells, in which the cornification process led to nucleus degradation and its DNA by specific DNAses [7,49]. The destruction of the nuclei in the outermost layer was verified on cryosections with Hoechst staining. The degradation of cellular DNA was also confirmed by the negligible iNos amplification by qPCR. This is consistent with the very low level of cellular DNA detectable in human corneocytes from washed hands rinses [50]. Although little DNA was extracted from corneocytes, the MDV ICP4 gene indicating the presence of the viral genome was easily detectable. Cells lytically infected with MDV contain the viral DNA in two forms, replicating in the nucleus (‘naked free’) and inside C capsids, located either in the nucleus or the cytoplasm. The fact that viral DNA was amplified from corneocytes in contrast to cellular DNA suggests that some viral DNA was protected from DNA lysis during cornification. The most suitable explanation of this result is that viral DNA was protected in capsids and resisted to DNAses, in contrast to cellular DNA. It is also interesting to mention that the viral load on total infected SEs decreased between D10 and D14, in contrast to the fluorescence linked to MDV infection, which remained stable. The most likely interpretation of these observations is that in this interval, a portion of infected cells became cornified and that the viral DNA present as ‘naked’ genomes in the nuclei was degraded at the same time as their cellular DNA. The presence of capsids in the cornified layer was also confirmed by TEM. This is the first time that MDV particles have been visualized in cornified cells, although their presence in the feather sheath has been suspected for a long time. In this study, we detected only capsids, with or without DNA (A, B and C capsids) and not enveloped virions. The absence of mature enveloped virions in cornified layers (in the stratum corneum) is intriguing, while a few seemed present in the underlying layers. Several interpretations are possible: (i) we failed to see them due to the compactness and high electron density of the cornified layers and/or to their low number; (ii) our model is incomplete and does not allow for their efficient production; (iii) these virions or their viral envelope were destroyed during cornification. Visualizing and quantifying virions using TEM remains very challenging *in situ*, particularly for viruses like MDV, which do not produce high litres. The implementation of real-time fluorescence imaging to track assembled capsids in order to study MDV morphogenesis is a new opportunity offered by this skin model, which could be developed in the future. Finally, the presence of viral genome and particles in corneocytes suggests that viral excretion may occur through these cells.

In our *in vitro* system, virions were demonstrated ultrastructurally, but it remains to be tested whether the virions are infectious and whether they are produced in a way that allows transmission. It is possible that corneocytes containing virions need to undergo further maturation induced by a dry environment [51], whereas our current model is maintained in a humid atmosphere. Dryness, mechanical stress or other factors may be required to release the virus from corneocytes. A feasible test of infectiousness would be to administer virion-containing SE material to chicks either via the respiratory route or intraperitoneally as done previously with poultry dust or dander [16,52]. Such an *in vivo* experiment would provide valuable insights into the relevance of this SE model in comparison with natural infection, and testing various treatments of the material may allow to define critical parameters of MDV transmission.

Although this 3D skin model is the most physiological one to date to study MDV infection of keratinocytes *in vitro*, it has some limitations. First, the primary keratinocytes used in this model are derived from leg skin epidermis, at the origin of scutate scales and not from FFE. Although both keratinocyte types lead to hard skin appendages, they undoubtedly exhibit differences, notably in the gene expression of corneous beta-proteins [53]. The second limitation of the present study is that it has not included histological comparisons with naturally infected skin or feather follicle tissue. However, the histology of infected tissues was reported previously [39,43, 54]. The structure and cell content of the SE differs from the feather follicle (FF) wall, which is infected by MDV. In the epidermis of the SE and of the FF wall, keratinocyte is the primary cell type of epidermis (>95%). In SEs, the number of cell layers in the epidermis is higher than in FF wall, showing four to six cell layers [42,55]; the FFE may contain additional cell types, although minor in quantity, such as melanocytes [56], feather stem cells [57] and possibly Langerhans cells. Note that the FF is surrounded by the dermis, containing fibroblasts as well as many other cell types absent from SEs. A third limitation is that the reproducibility and robustness of producing chicken SEs still need improvement in order to achieve more standardized products, similar to the progress made with human SEs over the past 20 years through the efforts of many contributors. A fourth limitation is the protocol of infection that we used by adding basal infected keratinocytes of the K8 cell line at the time of primary keratinocyte seeding. In chickens, it is thought that the FFE is infected through the entry of infected T-lymphocytes, although never experimentally demonstrated. Establishing an SE model infected in a more natural way will be a step forward in complexity, for example, by introducing latently or reactivated MDV-infected T-lymphocytes in the epidermis or the dermis. Protocols developed for 3D immunocompetent human skin models could serve for such purpose [58,59]. The refinement of the 3D chicken skin model in an immunocompetent system could also enhance to study the skin responses to MDV infection and other pathogens.

In summary, we present the first successful infection and replication of a pathogen, MDV, in an organotypic 3D chicken skin model reconstructed *in vitro*. MDV infection did not impair SE reconstruction. We confirmed the presence of virions in keratinocytes of suprabasal layers of the epidermis as shown earlier in FFE and observed for the first time viral particles in the cornified layer, from which dander originates, making this system a unique *in vitro* model to study MDV shedding.

## Abbreviations

ALI: air-liquid interface
A.U.: arbitrary unit
BAC: bacterial artificial chromosome
CESC: primary chicken embryonic skin cell
CPKe: embryonic chicken primary keratinocytes
FF: feather follicle
FFE: feather follicle epithelium
GaAHV-2: Gallid alphaherpesvirus 2
HBSS: Hanks Balanced Salt Solution
HPS: haematoxylin-phloxin-safranin
HVT: herpesvirus of turkey
KAT: Katushka fluorescent protein
KGM-2: Keratinocyte Growth Medium 2
KRT: keratin
MD: Marek’s disease
MDV: Marek’s disease virus
MMLV: Moloney murine leukaemia virus
qPCR: quantitative PCR
ROI: region of interest
SE: skin equivalent
TEM: transmission electron microscopy
VZV: varicella zoster virus
